# Betaine Improves Intestinal Functions by Enhancing Digestive Enzymes, Ameliorating Intestinal Morphology, and Enriching Intestinal Microbiota in High-salt stressed Rats

**DOI:** 10.3390/nu10070907

**Published:** 2018-07-16

**Authors:** Haichao Wang, Sisi Li, Shenglin Fang, Xiaojing Yang, Jie Feng

**Affiliations:** 1 Key Laboratory of Animal Nutrition & Feed Science, Zhejiang Province, College of Animal Sciences, Zhejiang University, Hangzhou 310058, China; qiusuoba@163.com (H.W.); lisisi@zju.edu.cn (S.L.); 11317015@zju.edu.cn (S.F.); 2 Department of Animal Science, College of Agricultural, Consumer and Environmental Science, University of Illinois at Urbana-Champaign, Urbana, IL 61801, USA; yang.teresaa@gmail.com

**Keywords:** betaine, high salt, osmoregulation, digestive enzymes, gut microbiota

## Abstract

To investigate the role of betaine in the intestinal functions of high-salt stressed rats, 32 four-week-old male Sprague–Dawley rats weighing 128.0 (SD 5.06) g were randomly allotted to four groups. The control group was fed with standard chow diet (0.4% NaCl), while the treatment groups were fed a high-salt diet (4.0% NaCl) supplemented with betaine at 0.0%, 0.5%, and 1.0%, respectively. The experiment lasted 28 days. The results showed that rats in the high-salt stressed groups had a significant increase in both water intake and kidney index (*p* < 0.05). The level of cortisol (COR) was increased in the high-salt stressed rats (*p* < 0.05), and returned to normal levels with betaine supplementation (*p* < 0.05). Aldosterone (ALD) was decreased in all high-salt diet groups (*p* < 0.05). Betaine supplementation decreased antidiuretic hormone (ADH) levels significantly (*p* < 0.05). High salt stress decreased the activities of amylase, lipase, trypsin, and chymotrypsin in the small intestinal luminal contents (*p* < 0.05), however, these activities increased with betaine supplementation (*p* < 0.05). The gut villus height of small intestine was significantly decreased in the high-salt diet group (*p* < 0.05). However, they were higher in the betaine supplementation groups than in the control group (*p* < 0.05). A similar result was observed in the ratio of villus height to crypt depth (*p* < 0.05). Both alpha diversity indexes and beta diversity indexes showed that high salt stress decreased the diversity of intestinal microbiota, while supplementation with betaine counteracted the negative effect. In conclusion, the results indicate that betaine improves intestinal function by enhancing the digestive enzymes, ameliorating intestinal morphology, and enriching intestinal microbiota of high-salt stressed rats.

## 1. Introduction

Betaine, also known as glycine betaine (*N*,*N*,*N*-trimethylglycine), is a small zwitterionic compound that is extensively distributed in organisms including bacteria, fungi, plants, invertebrates, and mammals [1]. Betaine is a significant component of many food and rich dietary sources include wheat bran (1339 mg/100 g), wheat germ (1240 mg/100 g), spinach (645 mg/100 g), beets (297 mg/100 g), pretzels (236 mg/100 g), and shrimp (219 mg/ 100 g) [2,3]. In mammals, betaine can be obtained not only by absorption through dietary intake, but also by endogenous synthesis from choline, and the primary role of betaine is to donate methyl group in one-carbon metabolism in the liver [1]. It is reported that betaine could alleviate fat accumulation in the liver [4]. Some reviews even recommend using betaine to treat nonalcoholic fatty liver disease (NAFLD) [5,6,7]. In the kidney, where the osmolarity fluctuates wildly because of urine concentration and dilution, betaine functions mainly as an osmolyte in the inner medulla to preserve the osmotic equilibrium and to maintain the tertiary structure and function of macromolecules [8]. Betaine also acts as an organic osmolyte in various life forms such as microorganisms [9,10], plants [11], and marine invertebrates [12]. In vitro studies also found that betaine could play a role in osmoregulation in mouse embryos [13], mouse hybridoma cells [14], rat liver sinusoidal epithelial cells [15,16], rat hepatic stellate cells [17], and chick embryo fibroblasts [18]. As a feed additive, betaine is widely used in animal science [19]. Most researchers consider betaine as a reliable source of methyl groups that can spare the role of choline and/or methionine in animal nutrition [20,21]. It has also been reported that betaine could alleviate the growth depression caused by coccidiosis or diarrhea [22,23]. Still, Ferket [24] found clear reductions in litter moisture and occurrence of diarrhea in turkeys supplemented with betaine and assumed that it was in association with the osmoregulation role of betaine. However, less information is documented on the osmoregulation role of betaine in nutrition. In the current experiment, we used a high-salt diet to create hyperosmotic conditions in order to investigate the role of betaine in intestinal functions and intestinal microbiota in rats.

## 2. Materials and Methods

### 2.1. Animals and Experimental Design

This work was approved by the Institutional Animal Ethics Committee of Zhejiang University. The experiment was carried out in the Laboratory Animal Center of Zhejiang University. All experimental procedures were undertaken with reference to the Guidelines for the Care and Use of Laboratory Animals in China. The Sprague–Dawley rats used in the investigation were purchased from the Laboratory Animal Center of Zhejiang Academy of Medical Sciences (Hangzhou, China). Thirty-two 4-week-old male Sprague–Dawley rats weighing 128.0 (SD 5.06) g were randomly allocated to four different groups, with eight rats in each group: A, basal diet (0.4% NaCl, 0.0% betaine) which was formulated in accordance with AIN-93G (American Institute of Nutrition) [25]; B, basal diet with modified sodium content (4.0% NaCl, 0.0% betaine); C, basal diet with modified sodium and betaine content (4.0% NaCl, 0.5% betaine); and D, basal diet with modified sodium and betaine content (4.0% NaCl, 1.0% betaine). The chow diets were specialized for our experiment by SLAC Experimental Animals Co., Ltd. (Shanghai, China). The betaine (99.4% purity) used in the present study was donated by the Healthy (Hangzhou) Husbandry Sci-tech Co., Ltd (Zhejiang, China). All animals were housed in a room with constant temperature (21 ± 1 °C) and humidity (75 ± 5%) under 12 h light/dark cycle. The rats were caged individually in stainless cages with free access to water and chow diet ad libitum throughout the entire feeding period. The experiment lasted 28 days. Body weight, feed intake, and water consumption were monitored twice a week. 

### 2.2. Sampling

On day 28, all rats were anesthetized with halothane. The plasma sample was collected from orbital blood (centrifuged at 3000× *g* for 10 min) and stored at −80 °C. The rats were then euthanized by cervical dislocation. Fresh livers and kidneys were weighed and collected separately. The samples were frozen in liquid nitrogen immediately. Intestinal contents from the duodenum, jejunum, ileum, and cecum were collected and rapidly frozen in liquid nitrogen. Segments of the duodenum, jejunum, and ileum were fixed in 10% formaldehyde for hematoxylin and eosin (H&E) staining. The mucosa of duodenum, jejunum, and ileum were scraped and then sharply frozen in liquid nitrogen. All snap-frozen samples in liquid nitrogen were stored at −80 °C for subsequent analysis.

### 2.3. Osmolarity and Electrolytes Assays

Pure deionized water was added to the samples of liver, kidney, intestinal digesta and mucosa (*v*/*w* = 9:1) separately and the suspensions were homogenized accordingly [26]. The osmolarity of the supernatants and plasma was determined by using the Fiske^®^ 210 freezing-point osmometer (Advanced Instruments, Norwood, MA, USA). The concentrations of sodium (Na) and potassium (K) were determined by using a flame photometer (ICE3100, Thermo Scientific, Waltham, MA, USA) and the concentrations of chloride (Cl) were measured by using a colorimetric procedure.

### 2.4. Plasma Endocrine Hormone Assays

Plasma aldosterone (ALD), cortisol (COR), and antidiuretic hormone (ADH) were measured by using commercially available kits (Jiancheng Bioengineering, Nanjing, China).

### 2.5. Digestive Enzymes Assays

Digestive enzymes activities (amylase, maltase, lipase, trypsin, and chymotrypsin) of the digesta from the duodenum, jejunum, and ileum were measured by using appropriate kits (Jiancheng Bioengineering, Nanjing, China).

### 2.6. DNA Isolation and 16S rRNA Gene Sequencing

Total bacterial genome DNA from caecal samples was extracted following the manufacturer’s instructions of the Qiagen DNA Extraction kit (Qiagen, Hilden, Germany). The quality and quantity of the isolated DNA were monitored on 1% agarose gels electrophoresis together with a NanoDrop ND-2000c UV-vis spectrophotometer (NanoDrop Technologies, Wilmington, NC, USA). 16S rRNA genes were amplified using barcoded primer set (341F: 5′-CCTAYGGGRBGCASCAG-3′, 806R: 5′-GGACTACNNGGGTATCTAAT-3′) covering the V3–V4 region. PCR amplification was carried out with Phusion^®^ High-Fidelity PCR Master Mix (New England Biolabs, Ipswich, MA, USA) and the protocols were summarized as follows: initial denaturation (94 °C for 3 min, 1 cycle), followed by 30 cycles of denaturation (94 °C for 45 s), annealing (62 °C for 30 s), and extension (70 °C for 45 s), with the last step at 72 °C for 5 min. The PCR products were purified using the GeneJET^TM^ Gel Extraction Kit (Thermo Scientific) and then quantified using QuantiFluor™ ST (Promega, Fitchburg, WI, USA). Sequencing libraries were generated using Ion Plus Fragment Library Kit 48 rxns (Thermo Scientific, USA) following manufacturer’s recommendations. The library quality was assessed by the Qubit@ 2.0 Fluorometer (Thermo Scientific, Waltham, MA, USA) and Agilent Bioanalyzer 2100 system. Finally, the library was sequenced on an Illumina HiSeq platform (San Diego, CA, USA), and generated ~250 bp paired-end reads.

### 2.7. Data Processing 

After quality control of the raw data by FastQC (version 0.11.3), paired-end reads were assigned to each sample based on their unique barcode and were then merged using FLASH (version 1.2.7) to obtain the raw tags [27]. Concatenated sequences were filtered out under specific filtering conditions [28] according to the QIIME software package (version 1.7.0) [29]. USEARCH software (version 6.1) was used to remove the chimeric sequences according to the UCHIME algorithm [30]. UPARSE software (version 7.0.1001) was used to cluster sequences into operational taxonomic units (OTUs) based on a 97% similarity threshold [31]. The phylogenetic affiliation of each 16S rRNA gene sequence was analyzed by the RDP Classifier (Version 2.2) algorithm [32] to annotate taxonomic information against the GreenGene database [33]. Alpha diversity indexes (the Observed species, Chao1, Shannon, and Simpson indexes) were calculated using the QIIME software package (version 1.7.0) and displayed with the R software package (version 2.15.3) to analyze the complexity of species diversity for a sample. Beta diversity indexes, including nonmetric multidimensional scaling (NMDS), unweighted unifrac distances, and linear discriminant analysis effect size (LEfSe) were used to evaluate differences of samples in species complexity. The NMDS ordination plot was measured according to Yue and Clayton [34], unweighted unifrac distances were calculated by QIIME software (Version 1.7.0), and LEfSe was performed using the vegan package of R software (version 2.15.3).

### 2.8. Statistical Analysis

Data analyses were performed using one-way ANOVA of the SAS 9.2 statistical software package (SAS Institute, Inc., Cary, NC, USA). The Duncan test was applied to multiple comparisons among groups, with significant differences being declared at *p* < 0.05.

## 3. Results

### 3.1. Growth Performance

From Table 1, we found that rats in high-salt stressed groups significantly increased their water intake (*p* < 0.05). The kidney index was also increased markedly in high-salt stressed rats (*p* < 0.05). There were no statistical differences in average daily feed intake (ADFI) or average daily gain (ADG) (*p* > 0.05).

### 3.2. Osmolarity and Electrolytes

As we can see from Table 2, there were no differences observed in the osmolarity of plasma, liver, kidney, luminal digesta, or mucosa of the small intestine among groups (*p* > 0.05). In each group, in terms of digesta osmolarity and mucosa osmolarity, the duodenum had the highest values and the ileum the lowest (*p* < 0.05). Table 3 showed that high salt stress decreased the Cl- content in plasma (*p* < 0.05), but it was increased in liver and kidney (*p* < 0.05). The contents of Na+ and K+ in plasma, kidney and liver were not different among groups (*p* > 0.05).

### 3.3. Endocrine Hormones

**Figure 1:**
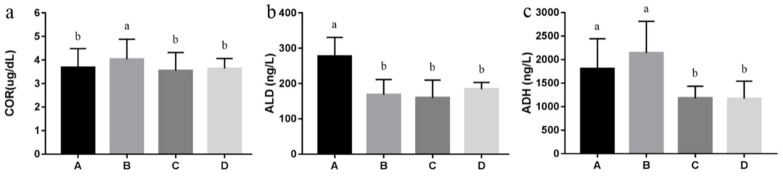
Effect of betaine on endocrine hormones of high-salt stressed Sprague–Dawley rats (*n* = 8 in each group). Note: ^a,b^ Values without common letters above the histogram differ significantly (*p* < 0.05). A: control group with standard diet (0.4% NaCl); B: high-salt diet (4.0% NaCl); C: high-salt diet (4.0% NaCl) with 0.5% betaine; D: high-salt diet (4.0% NaCl) with 1.0% betaine; (**a**) COR, cortisol; (**b**) ALD, aldosterone; (**c**) ADH, antidiuretic hormone.

As can be seen in Figure 1, the level of COR was increased in high-salt stressed rats (*p* < 0.05), and returned to normal level with betaine supplementation (*p* < 0.05). ALD was decreased in all high-salt diet groups (*p* < 0.05). ADH was increased in high-salt stressed rats (*p* > 0.05), when supplemented with betaine, it decreased significantly (*p* < 0.05).

### 3.4. Digestive Enzyme Activities

**Figure 2:**
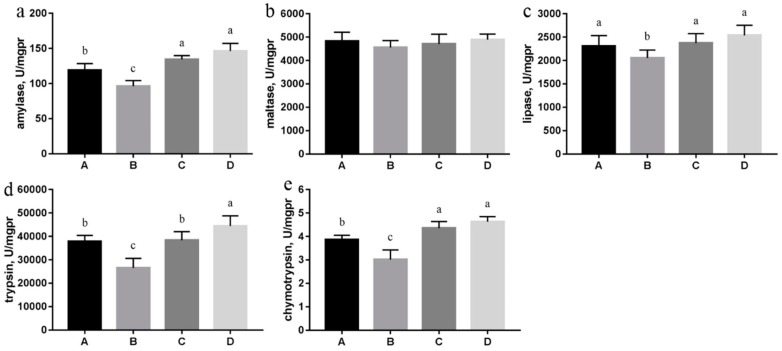
Effect of betaine on digestive enzyme activities of high-salt stressed Sprague–Dawley rats (*n* = 8 in each group). Note: ^a,b,c^ Values without common letters above the histogram differ significantly (*p* < 0.05). A: control group with standard diet (0.4% NaCl); B: high-salt diet (4.0% NaCl); C: high-salt diet (4.0% NaCl) with 0.5% betaine; D: high-salt diet (4.0% NaCl) with 1.0% betaine; (**a**) amylase; (**b**) maltase; (**c**) lipase; (**d**) trypsin; (**e**) chymotrypsin.

Data from Figure 2 showed that high salt stress decreased the activities of amylase, lipase, trypsin and chymotrypsin (*p* < 0.05). However, the activities of these enzymes markedly increased with betaine supplementation (*p* < 0.05). The data in trypsin activity showed that betaine supplementation at 1.0% was more powerful than at 0.5%.

### 3.5. Intestinal Morphology

**Figure 3:**
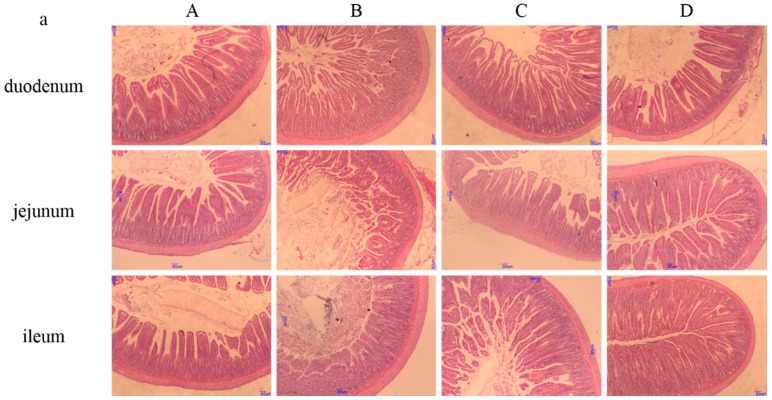

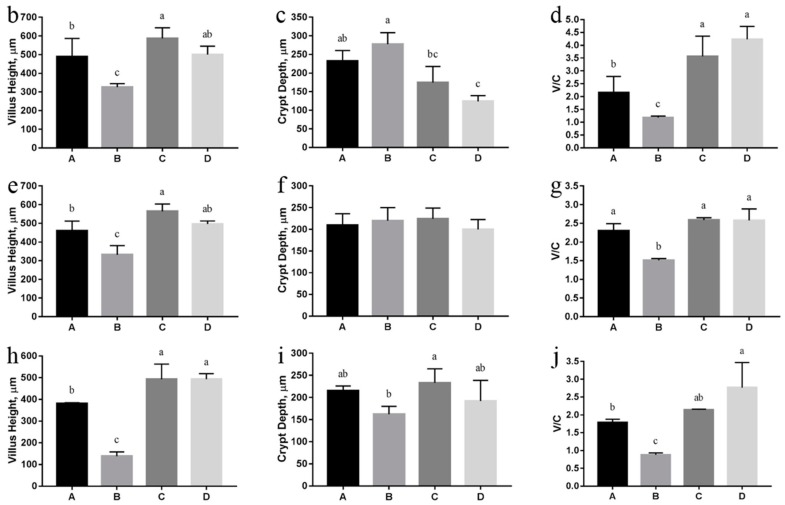
Histomorphology of small intestine between groups (*n* = 8 in each group). Note: ^a,b,c^ Values without common letters above the histogram differ significantly (*p* < 0.05). A: control group with standard diet (0.4% NaCl); B: high-salt diet (4.0% NaCl); C: high-salt diet (4.0% NaCl) with 0.5% betaine; D: high-salt diet (4.0% NaCl) with 1.0% betaine; (**a**): Hematoxylin and eosin (H&E)staining (100×); Duodenum villi height (**b**), crypt depth (**c**) and ratio of villus height to crypt depth (V/C) (**d**); Jejunum villi height (**e**), crypt depth (**f**) and V/C (**g**); Ileum villi height (**h**), crypt depth (**i**) and V/C (**j**).

Light micrographs of small intestinal histomorphology are displayed in Figure 3a. It can be seen that high salt stress disturbed the morphology of gut villi. When supplemented with betaine, the damaged villi were restored. This was verified with the statistical data (Figure 3). The gut villus heights of the duodenum, jejunum and ileum were all significantly decreased in high-salt diet group (*p* < 0.05). However, they were higher in the betaine supplementation groups than in the control group (*p* < 0.05). Similar results were observed in the ratio of villus height to crypt depth (*p* < 0.05).

### 3.6. Intestinal Microbiota

**Figure 4:**
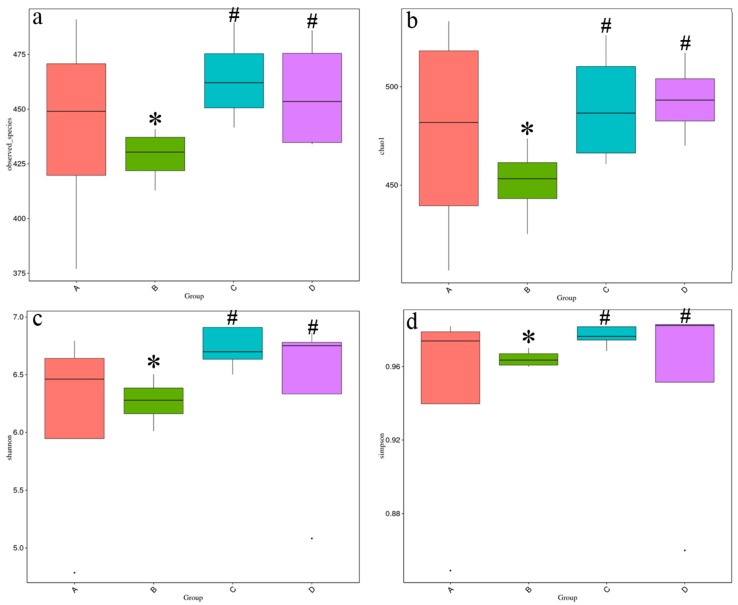
Alpha diversity of caecal microbiota between groups (*n* = 4 in each group). Note: * shows a statistical significant difference compared with the control group with standard diet (*p* < 0.05). ^#^ shows a statistical significant difference compared with the high-salt diet group (*p* < 0.05). A: control group with standard diet (0.4% NaCl); B: high-salt diet (4.0% NaCl); C: high-salt diet (4.0% NaCl) with 0.5% betaine; D: high-salt diet (4.0% NaCl) with 1.0% betaine; (**a**) Observed_species; (**b**) Chao1 index; (**c**) Shannon index; (**d**) Simpson index.

**Figure 5:**
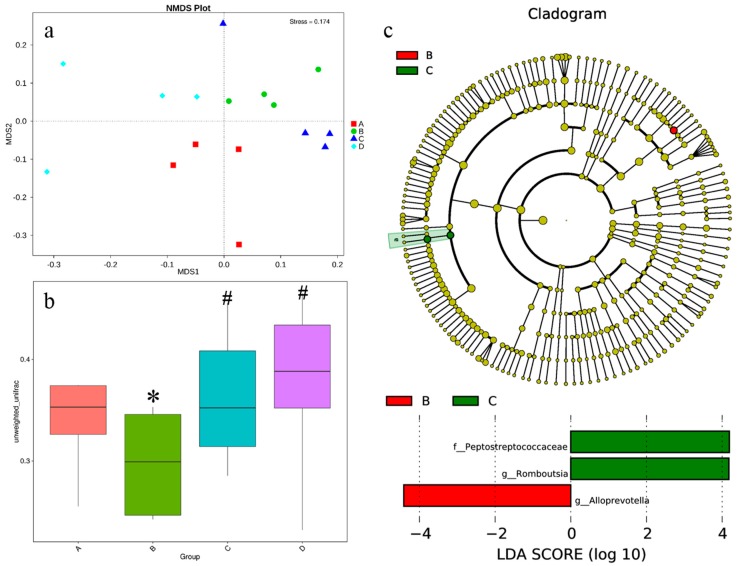
Beta diversity of caecal microbiota between groups (*n* = 4 in each group). Note: * shows a statistical significant difference compared with the control group with standard diet (*p* < 0.05). ^#^ shows a statistical significant difference compared with the high-salt diet group (*p* < 0.05). A: control group with standard diet (0.4% NaCl); B: high-salt diet (4.0% NaCl); C: high-salt diet (4.0% NaCl) with 0.5% betaine; D: high-salt diet (4.0% NaCl) with 1.0% betaine; (**a**) nonmetric multidimensional scaling (NMDS) plot; (**b**) unweighted_unifrac; (**c**) linear discriminant analysis effect size (LEfSe) analysis.

**Figure 6:**
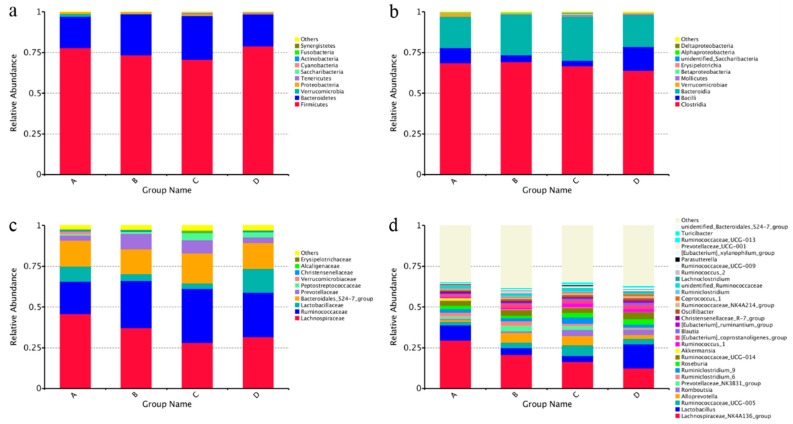
Caecal microbiota structure between groups (*n* = 4 in each group). Note: A: control group with standard diet (0.4% NaCl); B: high-salt diet (4.0% NaCl); C: high-salt diet (4.0% NaCl) with 0.5% betaine; D: high-salt diet (4.0% NaCl) with 1.0% betaine; (**a**) phylum level; (**b**) class level; (**c**) family level; (**d**) genus level.

Based on the high throughput sequencing, a total of 1,080,496 high-quality bacterial 16S rRNA sequences was obtained in the caecal chyme samples. Each sample yielded 67,531 sequences and 482 OTUs on average. There were fewer observed species in the high-salt stressed group than in other groups (Figure 4a). All of the alpha diversity indexes (Chao1, Shannon and Simpson indexes, Figure 4b–d) showed that high salt stress decreased the diversity of intestinal microbiota while supplementation with betaine could offset the decrease. In the NMDS plot (Figure 5a), samples from the same group had a closer distance than that between groups except that one sample in the high-salt diet supplemented with 0.5% betaine group was far away from its population. Unweighted unifrac distances were significantly altered by high salt stress (Figure 5b), which displayed a trend similar to alpha diversity indexes. LEfSe analysis showed that *Alloprevotella* had the greatest presence in group B, while *Peptostreptococcaceae* and *Romboutsia* were most present in group C (Figure 5c). The caecal microbiota structure is shown in Figure 6. At the phylum level (Figure 6a), the gut microbiota of all rats was dominated by Firmicutes, Bacteroidetes, Verrucomicrobia and Proteobacteria. At the class level (Figure 6b), the presence of Bacilli in group B and C was decreased, while that of Bacteroidia was increased as compared to group A and D. The presence of Verrucomicrobiae was decreased in all high-salt diet groups with or without betaine supplementation. At the family level (Figure 6c), the presence of *Ruminococcaceae* and *Prevotellaceae* in group B and C was increased while that of *Lactobacillaceae* was decreased as compared to group A and D. The presence of *Peptostreptococcaceae* was increased in both betaine supplementation groups. *Lachnospiraceae* and *Verrucomicrobiaceae* were decreased in all high-salt diet groups. At the genus level (Figure 6d), *Lactobacillus* in group B and C was decreased while *Alloprevotella* was increased as compared to group A and D. *Romboutsia* and *Ruminiclostridium_6* were increased in both betaine supplementation groups. *Lachnospiraceae_NK4A136_group* and *Akkermansia* were decreased in all high-salt diet groups.

## 4. Discussion

It has long been viewed that the maintenance of osmotic balance in response to high salt intake is a passive process that is mediated largely by increased water consumption to balance the salt load [35]. The feed intake was not affected by a high-salt diet in the current experiment, however, rats in high-salt diet groups greatly increased the intake of water. We also found high excretion of urine in high-salt diet groups. Deloof et al. [36] found that salt-loaded pregnant rats were able to increase their urine flow to excrete the entire sodium intake. They also demonstrated additional evidence that the concentration of sodium in plasma was similar between the salt-loaded rats and the control group. In our study, the sodium concentration in plasma was not different among groups, which also confirmed their results. Several reports [37,38,39,40,41,42] have shown that high salt intake leads to increased water intake and increased diuresis and/or natriuresis in rats, monkeys, and humans. 

Extracellular fluid volume homeostasis with regard to natriuretic regulation includes suppression of the renin–angiotensin–aldosterone System (RAAS), promotion of natriuresis, and reduction of renal nerve activities, all of which can concomitantly increase the excretion of urinary sodium and lead to an increase in the urine volume [43]. ALD is an essential mineralocorticoid hormone for sodium conservation. The level of ALD in our study was decreased in all high-salt diet groups, which also added proof that sodium was excreted in salt-loaded rats. Kitada et al. reported that high salt intake reprioritized osmolytes for body fluid conservation [43]. We found that there was an exchange of Cl- between plasma and visceral organs. The increasing water intake and urine excretion increased the burden on the kidneys. To maintain the balance of water and electrolyte metabolism, kidney developed a compensatory mechanism to support its function. This was verified by the increased kidney indexes of high-salt diet groups in our study. 

An increase in plasma osmolality due to high-salt intake activated hypothalamic osmoreceptors to stimulate ADH secretion by the posterior pituitary gland [44]. The resulting increase in the level of plasma ADH leads to an increase in renal water reabsorption and a decrease in water excretion. In our study, this was verified in that high salt intake markedly increased the concentration of ADH. In the betaine supplementation group, the concentration of ADH decreased sharply. We assumed that betaine maintained body water such that less ADH was released to reabsorb water in the kidney of high-salt stressed rats. Cortisol is regarded as one of the endocrine indicators of stress [45]. Severe chronic stress results in prolonged periods of high cortisol concentration. In our study, the concentration of cortisol was significantly increased in the high-salt diet group which indicated that rats in high-salt diet group were under stress. When supplemented with betaine, cortisol concentrations fell back to normal levels, which suggested that betaine could counteract the adverse conditions caused by high salt intake.

No differences were observed in the osmolarity of the plasma, liver, kidney, luminal digesta or mucosa of small intestine among the groups in the current study. Osmotic balance is one part of the internal environment homeostasis that is rigidly monitored by the system. Though we fed rats with high-salt diet, the change in osmolarity was regulated by the neuroendocrine system. Betaine may also play a role in the regulation of osmotic homeostasis. The fluctuation of the tested endocrine hormones may be proof of this assumption. The osmotic pressure of the intestinal contents varies during the process of digestion and absorption. In our study, we found that the osmolarity of the luminal contents and the gut mucosa gradually decreased along the gastrointestinal tract. We also observed that the osmolarity of the gut mucosa was higher than that in the luminal contents of each separate tract, except in the duodenum of the high-salt stressed group. It was assumed that the difference in osmotic pressure between the intestinal luminal fluid and the epithelium was necessary to control the osmosis inside the intestinal epithelial cells [46]. 

The digestive enzymes, which were fundamentally proteins, were disturbed by high salt stress in our experiment, however, they were revived by the addition of betaine in a dose-dependent manner. Pollard and Wyn Jones also found the protection of betaine against salt inhibition of enzymes [47]. In the fermentation of lysine, lactate, and pullulanase, betaine could increase the activity of key cellular enzymes so as to improve the production of these products [48,49,50]. Betaine, the so-called compatible solute, could interact with macromolecules without detrimental effect and could be up- and down-regulated without disturbing cellular functions. In contrast, inorganic ions, when present at high concentrations, can destabilize proteins and nucleic acids [51]. The effect of betaine involved universal water–solute–macromolecule interactions. Destabilizing agents such as ions could bind to proteins and caused them to unfold. However, betaine was excluded from biopolymer’s surface so that the protein folded more compactly, which was called the “osmophobic” effect [52]. With the presence of osmolytes, the native state of proteins was left free to function relatively unfettered by thermodynamic focus of the osmophobic force on the denatured state [53]. This was supported by a study that established a link between the effect of betaine on water structure and the ability to stabilize protein structure [54]. Pollard and Wyn Jones found that sequential N-methylation from glycine to betaine increased the effectiveness of protection [47]. They also noted that the protection may be limited to certain enzymes and generalizations may be inappropriate.

In our experiment, the gut villi were seriously affected by high salt stress. High salt decreased the length of villi and increased the thickness of crypts. When supplemented with betaine, the histomorphology of gut microstructure was greatly improved. The ratio of villus height to crypt depth also showed similar results. Kettunen et al. [55] also found that dietary betaine supplementation increased the epithelial villus–crypt ratio in bird gut and they thought the improved mucosal structure was due to both the methyl group donor nature and the osmotic nature of betaine. Moreover, Hooper found that gut microbiota also contributed to the development of the intestinal villi [56]. In our study, the developmental trend of intestinal microstructure was in line with the diversity of gut microbiota.

Because of the close relationship between intestinal microbial flora and the host in nutrient absorption, colonization resistance, immune function and so on, intestinal microbiota has received increasing attention in recent years and was even presumed as an essential organ [57]. In this study, we used high salt as a hazard element to test the effect of betaine on gut microbiota. The results showed that the diversity of cecum microbes was decreased in high-salt stressed group and that it could be recovered with the presence of betaine. LEfSe analysis showed that *Alloprevotella* levels were the highest in the high-salt stressed group, while *Peptostreptococcaceae* and *Romboutsia* were most present in the betaine-supplemented group. *Alloprevotella*, which is obligately anaerobic, non-motile, and Gram-negative, is weakly to moderately saccharolytic, and the major end products of fermentation are acetic and succinic acids [58]. *Romboutsia* is anaerobic and produces acetate and formate as end products of fermentation [59]. *Romboutsia* belongs to the family of *Peptostreptococcaceae* which is dominant in rat ileum microbiota [60]. 

Bielinska and coworkers found that a high-salt diet altered the intestinal bacteria composition and produced gut dysbiosis in rats [61]. It is reasonable that salt could produce a hyperosmotic environment to inhibit bacterial proliferation, and it has long been used as an antiseptic and food preservative. In fact, the ability of bacteria to adapt to changes in external osmolarity directly determines their survival rate and proliferation [62]. High salt intake may favor the propagation of some bacterial strains over others due to the difference in salt tolerance between bacterial strains [63]. We assumed that in the high-salt stressed environment, betaine could not only regulate the osmotic microenvironment for the microbes to survive, but also provide extra sources of carbon and nitrogen for the microbes to live and grow. We also observed that some microbes in 0.5% betaine group had a similar abundance to the high-salt stressed group, while others in 1.0% betaine group had a similar abundance to the control group. This may be due to the dose-dependent effect whereby the concentration of betaine at 0.5% was insufficient for the stressed microbes to recover while betaine at 1.0% concentration could enable the microbes to revive to the normal level.

## 5. Conclusions

In summary, as a potent organic osmolyte, betaine can cooperate with endocrine hormones to regulate water and electrolyte metabolism and could play a role in osmoregulation to permit cellular adaptation to adverse osmotic environments. Betaine improves intestinal functions by enhancing the digestive enzymes, ameliorating intestinal morphology, and enriching intestinal microbiota of high-salt stressed rats.

## Figures and Tables

**Table 1:** Effect of betaine on growth performance of high-salt stressed Sprague–Dawley rats.

Items	A	B	C	D	SEM	*p*
Initial weight (g)	128.4	128.1	127.9	127.8	0.90	0.9956
Final weight (g)	320.6	309.0	315.8	317.9	2.86	0.5424
ADG (g/day)	6.87	6.46	6.71	6.79	0.10	0.5562
ADFI (g/day)	24.77	26.32	26.74	26.07	0.46	0.5675
Water intake (mL/day)	32.74 ^b^	57.37 ^a^	58.99 ^a^	58.00 ^a^	4.16	0.0002
Liver index (%)	4.07	4.28	4.42	4.24	0.06	0.2475
Kidney index (%)	0.79 ^b^	0.91 ^a^	0.90 ^a^	0.88 ^a^	0.01	0.0160

Note: ^a,b^ Values without common superscript letters differ significantly (*p* < 0.05) (*n* = 8 in each group); A: control group with standard diet (0.4% NaCl); B: high-salt diet (4.0% NaCl); C: high-salt diet (4.0% NaCl) with 0.5% betaine; D: high-salt diet (4.0% NaCl) with 1.0% betaine; SEM: standard error of means; ADG: average daily gain; ADFI: average daily feed intake.

**Table 2:** Effect of betaine on osmolarity of high-salt stressed Sprague–Dawley rats (mOsm/kg).

Items	A	B	C	D	SEM	*p*
Plasma	336	329	338	333	1.2	0.8451
Liver	570	579	563	555	6.3	0.6042
Kidney	498	500	466	489	6.2	0.2071
Digesta						
Duodenum	612	650	570	605	16.6	0.3940
Jejunum	490	514	490	476	13.0	0.7976
Ileum	413	361	380	374	10.6	0.3752
Mucosa						
Duodenum	620	585	680	633	18.8	0.3823
Jejunum	520	548	560	505	15.4	0.6194
Ileum	475	493	518	528	24.4	0.8943

Note: A: control group with standard diet (0.4% NaCl); B: high-salt diet (4.0% NaCl); C: high-salt diet (4.0% NaCl) with 0.5% betaine; D: high-salt diet (4.0% NaCl) with 1.0% betaine; (*n* = 8 in each group). SEM: standard error of means.

**Table 3:** Effect of betaine on electrolytes of high-salt stressed Sprague–Dawley rats (mmol/L).

Items	A	B	C	D	SEM	*p*
Plasma						
Na	142.2	143.1	141.8	141.3	0.32	0.2730
K	5.75	5.82	5.84	5.95	0.09	0.8801
Cl	100.3 ^a^	99.8 ^ab^	98.5 ^bc^	97.9 ^c^	0.33	0.0174
Liver						
Na	98.6	99.0	101.9	101.4	1.15	0.6940
K	83.9	83.6	84.2	83.8	0.62	0.9901
Cl	118.6 ^b^	130.6 ^a^	134.3 ^a^	134.4 ^a^	1.74	0.0008
Kidney						
Na	119.6	124.6	126.3	121.3	1.38	0.3140
K	67.1	67.1	67.6	68.7	1.21	0.9668
Cl	97.9 ^b^	102.5 ^ab^	105.8 ^a^	104.4 ^a^	0.96	0.0150

Note: ^a,b,c^ Values without common superscript letters differ significantly (*p* < 0.05) (*n* = 8 in each group). A: control group with standard diet (0.4% NaCl); B: high-salt diet (4.0% NaCl); C: high-salt diet (4.0% NaCl) with 0.5% betaine; D: high-salt diet (4.0% NaCl) with 1.0% betaine; SEM: standard error of means; Na: sodium; K: potassium; Cl: chloride.
